# Functional Connectivity and MRI Radiomics Biomarkers of Cognitive and Brain Reserve in Post-Stroke Cognitive Impairment Prediction—A Study Protocol

**DOI:** 10.3390/life15010131

**Published:** 2025-01-20

**Authors:** Hanna Maria Dragoș, Adina Stan, Livia Livinț Popa, Roxana Pintican, Diana Feier, Nicu Cătălin Drăghici, Dragoș-Cătălin Jianu, Diana Chira, Ștefan Strilciuc, Dafin F. Mureșanu

**Affiliations:** 1 Department of Neurosciences, Iuliu Hațieganu University of Medicine and Pharmacy, No. 8 Victor Babeș Street, 400012 Cluj-Napoca, Romania; hanna_dragos2006@yahoo.com (H.M.D.); livia.popa@umfcluj.ro (L.L.P.); nicu.draghici@umfcluj.ro (N.C.D.); diana.chira@brainscience.ro (D.C.); stefan.strilciuc@brainscience.ro (Ș.S.); dafinm@ssnn.ro (D.F.M.); 2 RoNeuro Institute for Neurological Research and Diagnostic, No. 37 Mircea Eliade Street, 400364 Cluj-Napoca, Romania; 3 Neurology Department, Emergency Clinical County Hospital, No. 43 Victor Babes Street, 400347 Cluj-Napoca, Romania; 4 Department of Radiology, Iuliu Hatieganu University of Medicine and Pharmacy, No. 3–5, Clinicilor Street, 400006 Cluj-Napoca, Romania; roxana.pintican@gmail.com (R.P.); diana.feier@elearn.umfcluj.ro (D.F.); 5 IMOGEN Institute, Centre of Advanced Research Studies, Emergency Clinical County Hospital Cluj, 400347 Cluj-Napoca, Romania; 6 First Division of Neurology, Department of Neurosciences-VIII, “Victor Babes” University of Medicine and Pharmacy, E. Murgu Sq., No. 2, 300041 Timisoara, Romania; jianu.dragos@umft.ro; 7 Advanced Centre for Cognitive Research in Neuropsychiatric Pathology (NeuroPsy-Cog), Department of Neurosciences-VIII, “Victor Babes” University of Medicine and Pharmacy, 156 L. Rebreanu Ave., 300736 Timisoara, Romania; 8 First Department of Neurology, “Pius Brînzeu” Emergency County Hospital, 156 L. Rebreanu Ave., 300736 Timisoara, Romania; 9 Research Center for Functional Genomics, Biomedicine and Translational Medicine, Iuliu Haţieganu University of Medicine and Pharmacy, 32-38 Gheorghe Marinescu St., 400347 Cluj-Napoca, Romania

**Keywords:** post-stroke cognitive impairment, brain reserve, cognitive reserve, functional connectivity, MRI radiomics, quantitative EEG

## Abstract

Acute ischemic stroke (AIS) is frequently associated with long-term post-stroke cognitive impairment (PSCI) and dementia. While the mechanisms behind PSCI are not fully understood, the brain and cognitive reserve concepts are topics of ongoing research exploring the ability of individuals to maintain intact cognitive performance despite ischemic injuries. Brain reserve refers to the brain’s structural capacity to compensate for damage, with markers like hippocampal atrophy and white matter lesions indicating reduced reserve. Cognitive reserve involves the brain’s ability to optimize performance and use alternative networks to maintain function. Advanced methods of MRI and EEG processing may better assess brain reserve and cognitive reserve, with emerging predictive models integrating these measures to improve PSCI prediction. This article provides the design of a hospital-based study investigating the predictive role of functional connectivity and MRI radiomics in assessing PSCI occurrence one year after AIS. One hundred forty-four patients will be enrolled following strict inclusion/exclusion criteria. The patients will undergo comprehensive assessments, including neuropsychological testing, brain MRI, and quantitative EEG (QEEG), across four visits over a year. The primary outcome will be PSCI occurrence, and it will be assessed at six and twelve months after AIS. Secondary outcomes will include PSCI severity, recurrent AIS, and mortality. Statistical analyses will be performed to identify predictive factors using Cox proportional hazards models, and predictive models based on QEEG, MRI radiomics, and clinical data will be built. Early detection of AIS patients prone to developing PSCI might outline more effective therapeutic approaches, reducing the social and economic burden of ischemic stroke.

## 1. Introduction

Post-stroke cognitive impairment (PSCI) occurs in about 20% of acute ischemic stroke (AIS) patients within the first months after the acute event, and 38% of AIS patients develop PSCI after one year. PSCI is associated with poor long-term prognoses and poor quality of life [1,2,3]. The mechanisms involved in the pathogenesis of PSCI are insufficiently understood [4]. Predictive models using clinical and imaging biomarkers have failed to explain interindividual differences in PSCI occurrence [5,6,7,8,9,10].

The concepts of brain reserve and cognitive reserve have been used to explain why some patients with extensive AIS lesions exhibit only mild neurological and cognitive deficits while patients with isolated lesions may experience significant impairments [11,12]. Brain reserve is commonly conceived as neurobiological capital, implying that individual variation in the structural features of the brain allows some patients to better adapt to acute brain lesions [13]. It has been suggested that hippocampal atrophy, increased lateral ventricle volume, white matter lesions, and decreased cortical thickness might indicate reduced compensatory cerebral capacity and serve as markers of impaired brain reserve [7,14,15,16].

Magnetic resonance imaging (MRI) radiomics assesses the heterogeneity of brain lesions and subtle regional changes, considering voxel intensity, the distances between voxels, and spatial relationships. Radiomics-based texture analysis showed that the occurrence of PSCI was associated with atrophy and structural changes in the hippocampus and entorhinal cortex [17]. High values of AIS lesion entropy were correlated with AIS severity and poor prognosis [18]. Radiomics has been integrated into machine learning algorithms to predict brain age and correlate these structural abnormalities with post-stroke outcomes [19,20].

Cognitive reserve is an active model of reserve consisting of the brain’s ability to optimize cognitive performance and use alternative cognitive strategies to adapt to acute brain lesions [13,21]. Cognitive reserve exerts a protective effect on cognition by influencing brain reserve [22]. High cognitive reserve means an increased capacity for the brain to engage alternative remote neuronal networks and preserve brain function despite local structural damage [23]. This hypothesis has been addressed using functional connectivity analysis. Functional connectivity is defined as the temporal dependency of neuronal activation patterns of remote brain regions and can be assessed using quantitative electroencephalography (QEEG) [24,25,26]. AIS lesions seem to cause changes in low-frequency resting-state networks, resulting in increased abnormal local connectivity, which is associated with global disconnection and disruption of subcortical–cortical or cortico-cortical interactions [27,28]. The most investigated QEEG parameters in PSCI are spectral power, connectivity, and network analysis [27,29,30]. Also, psychological assessment tools such as the Cognitive Reserve Index questionnaire are being explored to quantify cognitive reserve. Patients with higher scores on the Cognitive Reserve Index questionnaire seem to have a lower incidence of PSCI [31,32].

**Figure 1:**
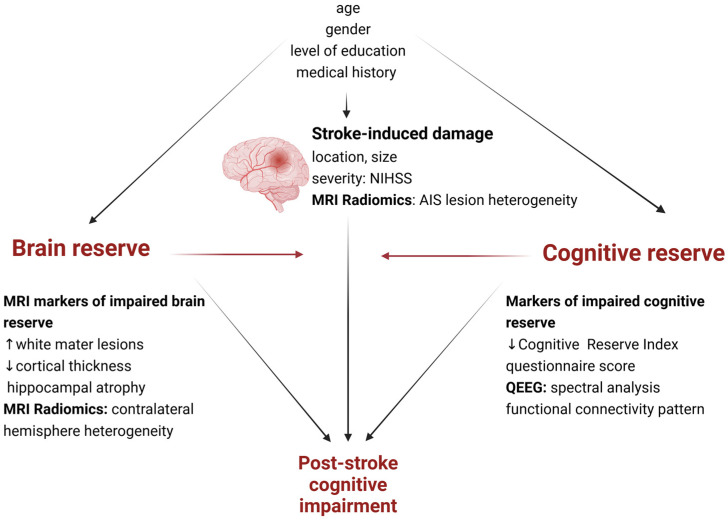
A quantitative approach to PSCI prediction based on clinical factors and potential markers of brain and cognitive reserves. Created in BioRender. Adapted from Umarova et al., 2021 [33].

A quantitative approach to PSCI is needed, considering the concepts of the brain and cognitive reserves assessed by connectivity and radiomics analysis (Figure 1). We hypothesize that predictive models that integrate functional connectivity patterns of cognitive reserve and MRI radiomics markers of brain reserve can better predict the occurrence of cognitive impairment (CI) one year after AIS. The primary objectives of this study are as follows:

1. To assess the predictive role of functional connectivity patterns in PSCI occurrence six months and one year after AIS;
2. To assess the predictive role of MRI radiomics features in PSCI occurrence six months and one year after AIS;
3. To develop efficient predictive models of PSCI occurrence one year after AIS based on functional connectivity and MRI radiomics.

## 2. Materials and Methods

This protocol is intended for an observational hospital-based cohort study of AIS patients. One hundred forty-four patients will be included. Considering a significance level of *p* = 0.05 with Z_α/2_ = 1.96; a statistical power of 80% with Z_β_ = 0.84; a 1:1 group ratio; and a 38% prevalence of PSCI in the first year after AIS [34], similar to other PSCI studies [1] and published protocols [7,35,36], and assuming a 20% drop-out rate, the sample size [37] was estimated to be 72 patients for each group (no PSCI and PSCI).

AIS will be defined as an acute focal neurological deficit confirmed by acute ischemic lesions on MRI according to the American Heart Association/American Stroke Association guideline [38,39]. Patients with prior cognitive impairment (CI) or dementia will be excluded. Prior CI will be defined by a summed score greater than 54.4 (corresponding to an average score > 3.4) on the short version of the Informant Questionnaire on Cognitive Decline in the Elderly (IQCODE) [40,41,42]. Also, patients with CI or dementia due to a strategic index stroke will be excluded. Strategic strokes are cerebral infarcts that can lead to PSCI or dementia independent of any other factor [43]. The strategic locations are the thalami, left angular gyrus, left capsular genu, right parietal lobe, left inferomesial temporal lobe, and left mesiofrontal lobe [43,44,45,46]. The inclusion and exclusion criteria are depicted below:

*Inclusion Criteria:*

- Patients ≥ 18 years with symptomatic supratentorial AIS confirmed by brain MRI;
- Patients with no significant pre-stroke functional disability (a modified Rankin Scale (mRS) score of 0 or 1);
- Patients without a history of CI or dementia (an IQCODE score of ≤3.4).

*Exclusion Criteria:*

- Patients with a prior symptomatic ischemic stroke or intracranial hemorrhage;
- Patients with CI or dementia due to a strategic index stroke;
- Patients with a history of chronic neurological disease;
- Patients with a history of chronic psychiatric illness;
- Patients with a history of harmful alcohol or drug use;
- Patients with pre-existing medical conditions such as cardiac, pulmonary, gastrointestinal, renal, or oncological diseases with a life expectancy of ≤one year;
- Patients with severe aphasia with a Goodglass and Kaplan score < 2 or a score on item 9 of the National Institute of Health Stroke Scale (NIHSS) ≥ 2;
- Patients with brain MRI or EEG contraindications.

The participants will be followed for one year with a total of four visits. The first visit will occur within 72 h after AIS onset. The second, third, and fourth visits will occur one month, six months, and one year after AIS. QEEG and brain MRI will be performed at baseline using specific protocols. The assessments that will be performed at every visit are displayed in Table 1.

The medical records of potential participants will be assessed for eligibility. Demographic and medical history data will be registered. The Cognitive Reserve Index questionnaire is a tool that quantifies cognitive reserve considering education level, working activity, and leisure time, and it will be applied at baseline [59]. The National Institute of Health Stroke Scale (NIHSS) [60] will be administered at every visit, while the mRS will be used at visits two, three, and four. The Mini-Mental State Examination (MMSE) and Montreal Cognitive Assessment (MoCA) will be applied to assess global cognitive domains [47,48,50,61]. Multiple neuropsychological tests have been chosen to increase the sensitivity of CI detection, covering a broad range of cognitive domains (Table 1). Neuropsychological examinations are unreliable in the acute phase of AIS, as the patient is likely to be subject to rapid fluctuations in several factors, such as their levels of consciousness, pain, fatigue, and distress [6,62]. Thus, these assessments will be performed one month, six months, and one year after AIS. Also, the Hospital Anxiety and Depression Scale (HADS) [57] and the 5-level EQ-5D quality of life scale (5Q-5D-5L) will be applied to assess mood disorders and quality of life [58].

### 2.1. Neuroimaging Assessment

All patients will receive a non-contrast brain MRI within ten days after AIS onset. The protocol consists of the following sequences: axial T2, axial Fluid-Attenuated Inversion Recovery (FLAIR), coronal FLAIR, axial Diffusion-Weighted Imaging (DWI)/Apparent Diffusion Coefficient (ADC), sagittal T1, and Susceptibility-weighted imaging (SWI). All MRI scans will be assessed for radiological markers of small vessel disease according to the Standards for Reporting Vascular Changes on Neuroimaging (STRIVE) classification system [63]: white matter lesions and Fazekas score [64,65], old asymptomatic lacunar infarcts [63], cerebral microbleeds [63,66], and enlarged perivascular spaces [63,67]. The size and volume of the AIS will be computed using 3D Slicer 5.6.2. software [68,69]. If the preliminary results show a significant correlation between AIS volume and PSCI occurrence, stratifying patients based on lesion volume (e.g., small, medium, and large according to the size of the affected middle cerebral artery territory) or including this feature as a covariate in the model could address data heterogeneity. The radiomics analysis workflow consists of four specific steps [70,71]: segmentation of the region of interest (the AIS lesion), radiomics feature extraction using PyRadiomics 2.2.0. software, feature selection, and development of clinical–radiomic prediction scores. According to recent studies [17] investigating MRI radiomics in PSCI patients, the following first- and second-order statistical features will be assessed: the mean gray level, the standard deviation of the gray levels, kurtosis, skewness, homogeneity, contrast, entropy, correlation, variance, the sum average, and the inverse difference moment. The brain MRI and radiomics analysis protocol is depicted in the Supplementary Material S1.

### 2.2. Electrophysiological Assessment

Resting-state EEG will be recorded within ten days after AIS onset using Neuron-Neuron-Spectrum-5 software. Nineteen Ag/AgCl electrodes will be applied according to the International 10–20 system. EEG activation techniques such as hyperventilation and intermittent photic stimulation will be applied according to specific protocols. Digital EEG data will be pre-processed and analyzed using Brain Vision Analyzer version 2.1. Artifacts will be removed both manually and using Independent Component Analysis [72]. The Fast Fourier Transform will be used to compute spectral power values. Absolute power will be summed across the following frequency bands: delta (0.5–4 Hz), theta (4–8 Hz), alpha (8–12 Hz), and beta (12–16 Hz) [73]. Also, the relative power [74,75] and the delta/alpha ratio [30] will be calculated. Connectivity markers such as correlation, cross-correlation, coherence, and the phase-locking value will be computed after segmentation and applying the Fast Fourier Transform [76,77,78]. The EEG acquisition and QEEG analysis protocol is depicted in the Supplementary Material S2.

### 2.3. Primary and Secondary Outcomes

The primary outcome will be the occurrence of PSCI, and the secondary outcomes will be PSCI severity, recurrent AIS, and mortality. All subjects will be classified into the following categories according to the Diagnostic and Statistical Manual of Mental Disorders, version 5 (DSM-5): no PSCI, mild PSCI, and major PSCI [79].

### 2.4. Statistical Analysis

Categorical variables will be presented as absolute numbers and percentages. The distribution of the continuous data will be determined. Non-normal continuous variables will be presented as medians or interquartile ranges, and non-parametric tests will be used. The Chi-square or Fisher’s exact tests will assess the associations between categorical variables. Spearman correlation coefficients will determine the associations between radiomics or QEEG features and neuropsychological scores. The types of variables are depicted in the Supplementary Material S3. An initial univariate analysis will assess the relationship between each variable (e.g., QEEG and MRI radiomics) and the outcome (PSCI). The variables showing significant associations (*p*-values < 0.05) will be included in a Cox proportional hazards regression.

As it is one of the most frequently used techniques in dementia risk prediction [80], a Cox proportional hazards regression will be used to identify predictive factors for PSCI. The aim of the Cox proportional hazards regression will be to model the simultaneous effects of multiple factors on the outcome [80]. The regression will be conducted to evaluate the associations between QEEG or MRI features (predictors) and neuropsychological test scores (outcomes) while also accounting for clinical factors as covariates (age, gender, the initial NIHSS score, and the discharge mRS score). All statistical analyses will be conducted with the R software package 4.4.2. The significance level will be alpha = 0.05.

A support vector machine (SVM) classification algorithm will be used to build a predictive model for PSCI occurrence. An SVM is a regression and clustering method that constructs models by integrating multiple variables that cannot be linearly separated. The model will be built using clinical factors (age, gender, the initial NIHSS score, and the discharge mRS score), radiomics, and QEEG features that will have been shown to have statistically significant associations with PSCI occurrence in the previous analysis. Classification will involve feature selection and leave-one-out cross-validation. A dimensionality reduction strategy (principal component analysis) will be applied to select the best features and limit the number of model variables and overfitting. Classification will be performed for the following comparisons: patients without PSCI versus mild PSCI patients, patients without PSCI versus major PSCI patients, and patients with mild PSCI versus major PSCI patients. The confirmed clinical diagnoses of the patients will further validate the accuracy of the SVM-based classification. The overall accuracy of the classification algorithm will be expressed as the mean +/− the SD. Its ability to predict PSCI will be assessed using the area under the curve. The stages of the classification algorithm are depicted in the Supplementary Material S4. The LibSVM open-source library will be used.

This study will be conducted according to the Declaration of Helsinki and was approved by the local ethics committee.

## 3. Discussion

This article provides the design of a hospital-based study that will investigate advanced MRI and EEG methods for PSCI prediction.

Recent studies [6,7,35] have used MRI and EEG methods to investigate risk factors associated with PSCI. The Tel-Aviv Brain Acute Stroke Cohort (TABASCO) study showed that low gray matter volume, low frontal cortex volume, high white matter lesion volume, and high cerebrospinal fluid volume predicted the development of PSCI with a dose-dependent relationship (*p* = 0.001) in patients with their first-ever AIS [81]. Moreover, white matter lesions and gray matter volumes were associated with executive function, attention, and the global cognitive score, while frontal cortex thickness was significantly correlated with memory [81]. Betrouni showed that radiomics features in the hippocampus and entorhinal cortex were significantly different between patients with PSCI and those without and were weakly but significantly correlated with MoCA and MMSE scores [17]. Moreover, together with clinical factors, radiomics features were used to train a machine learning model for PSCI prediction, achieving a global accuracy of approximately 88% [17]. Recent results suggest that MRI radiomics analysis might have better predictive performance than MRI volume measurements, showing signs of anatomical damage in infarct-free structures involved in cognitive function [17,82]. Thus, one of the objectives of our study will be to assess if advanced MRI techniques, such as radiomics, may better predict PSCI in specific cognitive domains.

Bournonville demonstrated lower functional network connectivity in PSCI patients, showing that a focal infarct can affect connectivity, even in remote regions, and can result in impairment of the whole-brain network [83]. Functional disconnections between the left superior and medial frontal gyri were associated with executive dysfunction, and functional disconnections between the left medial temporal gyrus, left inferior parietal lobe, left medial frontal gyrus, and posterior cingulum gyrus were associated with attention impairment [83].

These results showing functional connectivity and MRI radiomics patterns in PSCI highlight the need for further studies to validate these findings in different populations or identify new patterns and potential PSCI mechanisms. Existing CI risk prediction models built using the general population were applied to stroke patients, but their predictive performance was not ideal [84]. Thus, researchers have shifted their focus to machine learning [85,86]. In the last few years, machine learning algorithms based on neuroimaging and electrophysiological features have been increasingly applied to stroke research [87]. A recent meta-analysis of twenty-one studies showed that machine learning algorithms may be ideal tools for predicting PSCI, as a training set achieved a c-index of 0.82 (95% CI: 0.77–0.87) and sensitivity and specificity values > 70% [85].

Our study protocol has limitations. Firstly, this is a study protocol for a hospital-based cohort of AIS patients, resulting in predictive models with limited applicability and generalizability. As such, internal validation in other AIS cohorts from our region and external validation in distinctive AIS cohorts from different areas are intended. Secondly, including AIS patients with diverse AIS lesion features (location, size, volume, and severity) may introduce potential data heterogeneity, affecting the performance of the predictive model. Exclusion criteria, such as patients with strategic infarcts and prior symptomatic ischemic strokes, could address this issue. Moreover, depending on the preliminary results, stratifying the patients based on lesion features (cerebral territory and severity) or including these features as covariates in the model could reduce the effects of data heterogeneity. The SVM classification algorithm can be highly effective in predicting PSCI due to its ability to handle complex, non-linear relationships and high-dimensional datasets, but its limited interpretability may be a challenge in clinical decision-making [88,89]. To address this, incorporating Explainable AI techniques may support clinicians in understanding which biomarkers or clinical factors are most influential in predicting PSCI, bridging the gap between machine learning outputs and clinical decisions [89]. For instance, SHAP (Shapley Additive exPlanations) values could identify the impacts of radiomics or QEEG features on the predicted risk of PSCI.

Cognitive function is a leading factor affecting the potential rehabilitation prospects of stroke survivors. Timely detection of PSCI has profound implications for patients and the community, enabling better outcomes and reducing societal burden. Early diagnosis of PSCI allows timely interventions (e.g., cognitive rehabilitation, lifestyle changes, or medications) to slow down or potentially prevent further significant cognitive decline, improving patients’ overall quality of life and independence in daily activities [90]. Moreover, when PSCI is detected early, patients can receive personalized rehabilitation programs that target specific deficits, such as memory, attention, or executive function. Also, early diagnosis of PSCI helps patients and families plan for the future, including managing rehabilitation expectations, making informed care decisions, and ensuring the necessary resources and support systems.

Early detection of PSCI can lead to broader societal benefits. CI often shifts the responsibility of care to family members or caregivers. Early diagnosis of PSCI allows early interventions, potentially delaying severe CI and reducing caregivers’ burden. PSCI increases the risk of institutionalization and long-term care [91], but timely diagnosis can lead to cost-effective interventions that prevent or delay these outcomes, reducing overall healthcare expenditures. For younger stroke survivors who are still in the workforce, addressing cognitive deficits early can aid in their rehabilitation and return to work, contributing positively to society and reducing economic losses.

To date, there is no predictive score, including clinical, imaging, and electrophysiological features, that can be used to identify stroke survivors who are prone to developing PSCI. Furthermore, no study has used both EEG functional connectivity and quantitative brain MRI techniques to measure CI risk after AIS in the brain and cognitive reserve paradigm. A quantitative approach to assess interactions between different risk factors from the clinical, electrophysiological, and neuroimaging fields would more accurately predict CI after ischemic stroke. Early detection of AIS patients prone to developing CI might outline new concepts for targeted prevention of PSCI and functional disability, reducing the social and economic burden of stroke.

## Figures and Tables

**Table 1:** Assessment schedule and data extraction.

	**Visit 1**	**Visit 2**	**Visit 3**	**Visit 4**
Demographic data	X			
Patient medical history	X			
Cognitive Reserve Index questionnaire	X			
Index AIS data: vascular territory, etiology	X			
National Institute of Health Stroke Scale (NIHSS)	X	X	X	X
Modified Rankin Scale (mRS)		X	X	X
IQCODE	X			
Goodglass and Kaplan Scale	X			
Global cognitive function assessment: The Mini-Mental State Examination (MMSE) [47,48,49] Montreal Cognitive Assessment (MoCA) [47,50]		X	X	X
Executive function assessment: Trial Making Test-A (TMT-A) [51] Verbal Fluency Test-Controlled Oral Word Association Test-CFL Version (VFT-CFL) [52] Digit Symbol Processing Speed Index, Wechsler Adult Intelligence Scale, 4th edition (DS-WPSI) [53]		X	X	X
Attention and visuospatial orientation: Stroop Color–Word Test (Stroop) [54]		X	X	X
Memory and learning: Digit Span Backward, Wechsler Adult Intelligence Scale, 4th edition (DS-BW) [55] Rey Auditory Verbal Learning Test (RAVLT) [56]		X	X	X
Hospital Anxiety and Depression Scale (HADS) [57]		X	X	X
5-level EQ-5D quality of life scale (5Q-5D-5L) [58]		X	X	X
EEG + QEEG protocol	X			
Brain MRI + radiomics protocol	X			

## Data Availability

Not applicable.

## Supplementary Materials

The following supporting information can be downloaded at https://www.mdpi.com/article/10.3390/life15010131/s1, Supplementary Material S1. Brain MRI acquisition and radiomics analysis protocol; Supplementary Material S2. EEG acquisition and QEEG analysis protocol; Supplementary Material S3. Table S1. Types of variables; Supplementary Material S4. Classification algorithm protocol.
