# Supplementary material for: Functional Connectivity and MRI Radiomics Biomarkers of Cognitive and Brain Reserve in Post-Stroke Cognitive Impairment Prediction—A Study Protocol
Source: Life (Basel). 2025 Jan 20;15(1):131. doi: 10.3390/life15010131 (PMC11767096; doi:10.3390/life15010131)
Supplement: Supplementary file 1 [file life-15-00131-s001.zip › life-3287952-supplementary.pdf]

SUPPLEMENTARY MATERIALS

Supplementary Material S1. Brain MRI acquisition and radiomics analysis protocol

All patients will perform a non-contrast brain MRI within the first ten days of AIS onset in a 1.5T GE scanner using an eight-channel head coil. The protocol consists of the following sequences: axial T2, axial Fluid-Attenuated Inversion Recovery (FLAIR), coronal FLAIR, axial Diffusion Weighted Imaging (DWI)/Apparent Diffusion Coefficient (ADC), sagittal T1, Susceptibility-weighted imaging (SWI).

A senior neuro-radiologist will diagnose AIS lesions based on the DWI images. The AIS lesion size and volume will be calculated using the 3D Slicer software.

Small vessel disease (SVD) radiological markers

MRI scans will be assessed for SVD radiological markers according to the Standards for Reporting Vascular Changes on Neuroimaging (STRIVE) classification system (1). The following markers will be extracted:

- white matter intensities graded using Fazekas score
- old lacunar infarcts defined as a round or ovoid, subcortical, fluid-filled (similar signal as CSF) cavity of between 3 mm and 15 mm in diameter on T2-weighted or T1-weighted, with corresponding central CSF-like hypointensity and a surrounding rim of hyperintensity on FLAIR images
- cerebral microbleeds defined as round hypointense lesions on SWI with a diameter < 10 mm and classified as lobar or deep
- enlarged perivascular spaces are defined as smooth margin, round, oval, or linear-shaped lesions, sized up to 3 mm, with a signal intensity equal to CSF on T2-weighted images.

Cortical thickness measurement

Cortical thickness measures the width of gray matter and seems related to synaptic density, synaptic pruning, and intracranial myelination. (2). The frontal, parietal, temporal, and occipital cortical thickness will be computed using T1-weighted images and FreeSurfer software (2,3).

MRI radiomics analysis (RA)

RA features will be extracted and calculated from MRI images. RA workflow consists of four steps (4):

- segmentation of the region of interest (AIS lesion, contralateral hemisphere corresponding region) on T2 and ADC sequences using 3D Slicer software
- extraction of radiomics features data using PyRadiomics software
- feature selection through univariate statistical analysis methods (Mann-Whitney test)
- developing a clinical-radiomic prediction tool by applying multivariate analysis methods.

RA encompasses all features that can be computed from an image, in addition to the size, shape, and surface of anatomical structures; first-order statistics are used to study the voxel value distribution without considering a spatial relationship, while second-order statistics describe the spatial relationship between the values (4,5). These are mainly computed through the co-occurrence matrix (6). According to recent studies (7) investigating MRI radiomics in PSCI patients, the following first-order statistics features will be used: mean grey level, standard deviation of grey levels, kurtosis, and skewness. Second-order texture values are derived from the co-occurrence matrix whose entries represent the number of times that intensity levels i and j occur in two voxels separated by the distance d in the direction a (7). Texture analysis features such as homogeneity, contrast, entropy, correlation, variance, sum average, and inverse difference moment will be assessed, similar to recent studies on RA in PSCI patients (7).

Supplementary Material S2. EEG acquisition and QEEG analysis protocol

Eyes closed and eyes open, resting-state EEG will be recorded within the first ten days after AIS onset using Neuron-Spectrum.NET software. Nineteen Ag/AgCl electrodes will be applied according to the International 10-20 system (Fp1, Fp2, F3, F4, C3, C4, P3, P4, O1, O2, F7, F8, T3, T4, T5, T6, Fz, Cz, Pz). A reference electrode will be placed between Fz and Cz, and a ground electrode will be positioned on the collarbone. Electrode impedances are between 5-10 Hz or less. EEG activation techniques such as hyperventilation and intermittent photic stimulation will be applied according to specific protocols. Conventional EEG screening will assess the dominant rhythm and the presence of epileptiform discharges.

QEEG pre-processing

The digitally recorded EEG data will be pre-processed and analyzed using Brain Vision Analyzer version 2.1. Semi-automatic raw data inspection will be used to remove artifacts, predefining an artifact removal interval starting 0.2 seconds before and ending 0.2 seconds after the artifact (8). The low-pass and high-pass filters will be set to 0.5 and 40 Hz, respectively, and the notch filter will be set to 50 Hz. The Independent Component Analysis method will be applied with the following parameters: kurtosis > 3 and energy > 2 to remove any remaining artifacts. The EEG signal will be segmented into 2-second epochs.

QEEG spectral analysis

The Fast Fourier Transform will be performed to compute spectral power values. Absolute power will be summed across the following frequency bands: delta (0.5-4 Hz), theta (4-8 Hz), alpha (8-12 Hz), and beta (12-16 Hz), according to the protocols of previous QEEG studies on PSCI patients (9). For each electrode, the following parameters will be calculated: relative power for the delta, theta, alpha, and beta bands, and delta/alpha ratio (DAR). Relative power for each frequency band will be computed as the ratio of absolute power for a given band to total power summed across the 0.5-16 Hz range (10). DAR is a ratio of absolute power values for delta and alpha bands (11). The peak alpha frequency (PAF) will be assessed as a measure of alpha slowing and is calculated by identifying the frequency corresponding to the maximum power values in the 5.62-12.45 Hz range according to the approach of previous studies on PSCI (10,12,13).

QEEG connectivity analysis

Connectivity transforms such as correlation, cross-correlation, and coherence provide the computation of functional connectivity measures based on a set of channel pairs within a connectivity matrix (14). Time-domain methods such as correlation and cross-correlation assess the functional interaction and information transfer between brain areas over time and will be computed following segmentation and windowing (14). Frequency-domain connectivity techniques such as coherence and phase-locking value seem to achieve better accuracy in extracting more relevant and robust functional connectivity features and will be computed following the Fast Fourier Transform (14). Coherence quantifies the cortico-cortical functional connectivity, and it is a sensitive measure that can detect prodromal changes in functional and effective connectivity (15).

Supplementary Material S3

Table S1. Types of variables

Data	Variable type
**Data**	**Variable type**
**Demographics**	
Age (years)	Continuous
Gender (male/female)	Dichotomous
Education level (years)	Continuous
Hand dominance (right/left/ambidextrous)	Categorical
**Medical history**	
Hypertension, diabetes, atrial fibrillation, hypercholesterolemia, prior myocardial infarction (yes/no)	Dichotomous
Smoking status (current/former/never)	Categorical
Pre-stroke cognitive status (IQCODE score)	Continuous
**Clinical features related to index stroke**	
Classification of AIS: TACS, PACS, POCS, LACS	Categorical
TOAST Classification: atherothrombotic, cardioembolic, small vessel disease, other determined causes, undetermined causes	Categorical
NIHSS at admission, at one, six, and twelve months	Continuous
mRS at discharge, at one, six, and twelve months	Continuous
Medical complications during admission: infections, seizures (yes/no)	Dichotomous
**Neuropsychological tests**	
MMSE, MoCA, Stroop, DS-BWI, DS-WPSI, TMT-A, VFT-CFL, RAVLT	Continuous
**Cognitive reserve markers:** CRIq	Continuous
**Imaging markers and radiomics features**	
Infarct volume, cortical thickness	Continuous
White matter hyperintensities, Fazekas score	Categorical
Lacunes (present/absent)	Dichotomous
Microbleeds (deep/superficial/absent)	Categorical
Perivascular spaces (basal ganglia/centrum semiovale/absent)	Categorical
MRI radiomics features	Continuous
**EEG and QEEG markers**	
Epileptiform activity (yes/no)	Dichotomous
**QEEG markers:** relative spectral powers (delta, theta, alpha, beta), alpha/beta ratio, asymmetry index, intra- and inter-hemispheric coherence, phase locking value, entropy	Continuous

TACS=total anterior circulation stroke, PACS=partial anterior circulation stroke, POCS=posterior circulation stroke, LACS=lacunar stroke

Supplementary Material S4. Classification algorithm protocol

A support vector machine (SVM) classification algorithm will be used to build a predictive model for PSCI occurrence. SVM is a regression and clustering method that constructs models by integrating multiple variables that cannot be linearly separated. The model will be built using clinical factors (age, gender, initial NIHSS score, discharge mRS score), radiomics, and QEEG features, which have been shown to have a statistically significant association with PSCI occurrence in the previous analysis.

Not all these features could be expected to contribute to the classification of the groups. A dimensionality reduction strategy will be applied based on principal component analysis to select the best predictors and to limit the number of model variables and overfitting.

1. Dataset preparation – input data and standardization (normalization or scaling so that all features are on a similar scale to optimize SVM performance)
2. Initialization – define SVM algorithm parameters: nonlinear Kernel function, regularization parameter C (controls the trade-off between maximizing the margin and minimizing classification error), Kernel parameters (e.g., gamma for RBF kernels)
3. Implement leave-one-out cross-validation (LOOCV)
   LOOCV involves splitting the dataset so that each instance serves as a test set exactly once, and the rest form the training set.
   For a dataset with n instances:
   1. Loop through all data points i=1,2, …, n:
      - Training Set: exclude the ith instance
      - Test Set: Use only the ith instance
   2. Train the SVM model using the n−1 training instances.
   3. Test the SVM model on the ith instance and store the prediction.
4. Model training
   For each iteration in LOOCV:
   - The SVM learns a hyperplane that separates the classes by maximizing the margin between the support vectors of different classes, considering the regularization parameter **C**.
   - Depending on the kernel, transformations may be applied to map the data to a higher-dimensional space where it becomes linearly separable.
5. Prediction - for each left-out test instance, the SVM uses the hyperplane learned during the training phase to predict its label
6. Evaluation:
   - After completing all n iterations, compare the predicted labels against the true labels for all test instances
   - Common evaluation metrics include accuracy, precision, recall and F1 score
7. Hyperparameter Tuning – repeat the LOOCV process with different combinations of SVM parameters (e.g., C, kernel type, gamma) to identify the configuration that optimizes performance metrics

The actual clinical diagnosis of patients will further validate the accuracy of SVM-based classification.

The global classification accuracy will be expressed as the mean ± SD. The predictive ability will be assessed using the area under the curve (AUC). The open-source library (www.csie.ntu.edu.tw/~cjlin/libsvm/) and Python will be used for algorithm development.

1. Wardlaw JM, Smith EE, Biessels GJ, Cordonnier C, Fazekas F, Frayne R, et al. Neuroimaging standards for research into small vessel disease and its contribution to ageing and neurodegeneration. The Lancet Neurology. 2013 Aug;12(8):822–38.
2. Tahedl M. Towards individualized cortical thickness assessment for clinical routine. Journal of Translational Medicine. 2020 Apr 3;18(1):151.
3. Fischl B. FreeSurfer. NeuroImage. 2012 Aug 15;62(2):774–81.
4. Chen Q, Xia T, Zhang M, Xia N, Liu J, Yang Y. Radiomics in Stroke Neuroimaging: Techniques, Applications, and Challenges. Aging Dis. 2021 Feb 1;12(1):143–54.
5. Lubner MG, Smith AD, Sandrasegaran K, Sahani DV, Pickhardt PJ. CT Texture Analysis: Definitions, Applications, Biologic Correlates, and Challenges. Radiographics. 2017;37(5):1483–503.
6. Haralick RM, Shanmugam K, Dinstein I. Textural Features for Image Classification. IEEE Transactions on Systems, Man, and Cybernetics. 1973 Nov;SMC-3(6):610–21.
7. Betrouni N, Jiang J, Duering M, Georgakis MK, Oestreich L, Sachdev PS, et al. Texture Features of Magnetic Resonance Images Predict Poststroke Cognitive Impairment: Validation in a Multicenter Study. Stroke. 2022 Jul 13;101161STROKEAHA122039732.
8. Popa LL, Iancu M, Livint G, Balea M, Dina C, Vacaras V, et al. N-Pep-12 supplementation after ischemic stroke positively impacts frequency domain QEEG. Neurol Sci. 2022 Feb;43(2):1115–25.
9. Muresanu DF, Alvarez XA, Moessler H, Novak PH, Stan A, Buzoianu A, et al. Persistence of the effects of Cerebrolysin on cognition and qEEG slowing in vascular dementia patients: Results of a 3-month extension study. Journal of the Neurological Sciences. 2010 Dec;299(1–2):179–83.
10. Schleiger E, Wong A, Read S, Rowland T, Finnigan S. Poststroke QEEG informs early prognostication of cognitive impairment. Psychophysiology. 2017 Feb;54(2):301–9.
11. Schleiger E, Sheikh N, Rowland T, Wong A, Read S, Finnigan S. Frontal EEG delta/alpha ratio and screening for post-stroke cognitive deficits: The power of four electrodes. International Journal of Psychophysiology. 2014 Oct 1;94(1):19–24.
12. Yuasa T, Maeda A, Higuchi S, Motohashi Y. Quantitative EEG data and comprehensive ADL (Activities of Daily Living) evaluation of stroke survivors residing in the community. J Physiol Anthropol Appl Human Sci. 2001 Jan;20(1):37–41.
13. Finnigan S, Robertson IH. Resting EEG theta power correlates with cognitive performance in healthy older adults. Psychophysiology. 2011 Aug;48(8):1083–7.
14. Chiarion G, Sparacino L, Antonacci Y, Faes L, Mesin L. Connectivity Analysis in EEG Data: A Tutorial Review of the State of the Art and Emerging Trends. Bioengineering (Basel). 2023 Mar 17;10(3):372.
15. Bowyer SM. Coherence a measure of the brain networks: past and present. Neuropsychiatric Electrophysiology. 2016 Jan 17;2(1):1.
